# Reduction of anxiety symptoms among women within a collaborative care model and women’s health settings

**DOI:** 10.1017/S1463423623000440

**Published:** 2023-12-04

**Authors:** Lindsay R. Standeven, Kristen N. Miller, Alissa Mallow, Roni Berger, Virna Little

**Affiliations:** 1 Department of Psychiatry and Behavioral Sciences, Johns Hopkins Reproductive Mental Health Center, Baltimore, MD, USA; 2 School of Medicine, Johns Hopkins University, Baltimore, MD, USA; 3 School of Social Work, Adelphi University, Poughkeepsie, NY, USA; 4 Concert Health, Inc., New York, NY, USA

**Keywords:** anxiety, collaborative care, evidence-based practice, primary care, symptom reduction, women’s health

## Abstract

**Aim:**

The purpose of this study is to focus on changes in anxiety symptoms among women treated in women’s health practices and under a collaborative care model.

**Background:**

Research on collaborative care has largely focused on improving depressive and anxiety symptoms among adults in primary care settings. The applicability of collaborative care in other healthcare settings is underreported with limited research investigating if collaborative care has advantages in subpopulations treated in both traditional primary care settings and other healthcare settings, such as women’s health practices.

**Methods:**

This study, completed through secondary data analysis of the electronic record of N = 219 women across three women’s healthcare centers, evaluated if instituting a collaborative care model is associated with reduced anxiety symptoms and which factors (eg, primary diagnosis, duration of care, and use of psychotropic medications) are associated with anxiety outcomes. Anxiety symptoms were assessed using the Generalized Anxiety Disorder 7-item scale (GAD-7) at entry into and at termination from collaborative care services.

**Results:**

Overall, there was a significant reduction in average anxiety scores from baseline to termination of collaborative care (*t*(218) = 12.41, *P* < 0.001). There was a main effect for the duration of time receiving collaborative care services on anxiety score reduction (β = −0.28, SE = 0.06, *P* < 0.001) with a significant reduction in anxiety symptoms at the 90-day mark (*t*(218) = 10.58, *P* < 0.001). Therefore, collaborative care can be useful in women’s health practices in reducing anxiety symptoms over a 90-day time period.

## Introduction

Although ‘worry’ and ‘anxiety’ may be common human experiences, it is postulated that approximately 31.6% of the adult population in the United States will experience a diagnosable anxiety disorder within their lifetime, with women being 1.5–2 times more likely to be diagnosed than men (McLean *et al.*, 2011; Kessler *et al.*, 2012; Jalnapurkar *et al.*, 2018). Often unaware that their symptoms are caused or worsened by anxiety, individuals may present to their care providers with symptoms not typically associated with medical diagnoses and/or primary medical condition(s) exacerbated by underlying anxiety (Brahmbhatt *et al.*, 2021).

While the importance of screening for anxiety in primary care is therefore paramount, anxiety remains underdiagnosed (Buszewicz and Chew-Graham, 2011). Notably, the Healthcare Effectiveness Data and Information Set (HEDIS) measures, frequently used in primary care practices, provide multiple screens for depression, but none for anxiety (National Committee for Quality Assurance, 2022). Although men are overall less likely to receive treatment than women, women were more likely to be treated for mental health conditions by their primary care providers, whereas men were more likely to be referred out to psychiatric/mental health practitioners (Wang *et al.*, 2005). Because practitioners in both primary care (eg, general internal medicine) and women’s health (eg, obstetric and gynecology) settings may not have the clinical time, training, or resources to address anxiety symptoms or disorders (eg, optimally titrate psychotropic medications or provide necessary therapeutic interventions), alternative behavioral health interventions to address mental health needs are needed.

Collaborative care is an evidence-based model which addresses behavioral health diagnosis (such as depression, anxiety, alcohol, or substance abuse) in primary care settings (AIMS Center, 2022). Supported by over 80 randomized studies, collaborative care in primary care practices is well-established (Unützer *et al.*, 2002; Gilbody *et al.*, 2006; Katon and Unützer, 2006; Katon *et al.*, 2012; Kroenke and Cheville, 2022); it is a pragmatic and cost-effective strategy to provide improved access to behavioral health care in primary care practices (Goodrich *et al.*, 2013). This patient-centered approach, which is led by the primary healthcare provider, includes a practice-dedicated behavioral care manager and psychiatric consultant all expertly trained to address symptoms of depression and anxiety (Katon and Unützer, 2006; Raney, 2015). Collaborative care typically includes psychoeducation and evidence-based mental health scales in combination with brief psychotherapeutic and/or psychotropic medication interventions (Goodrich *et al.*, 2013).

Research on collaborative care models to date has largely focused on the effectiveness of collaborative care services in addressing depression and anxiety symptoms (Gilbody *et al.*, 2006; Rubenstein *et al.*, 2010; Thota *et al.*, 2012; Muntingh *et al.*, 2016) and specifically in primary care settings (Unützer *et al.*, 2002; Bower *et al.*, 2006; Muntingh *et al.,* 2014). Although research on the effectiveness of collaborative care in treating depression among women and in other care settings has been expanding (Fairbrother *et al.*, 2016; Marcus *et al.* 2019; Terrazas *et al.*, 2019; Miller *et al.*, 2020), research on the effectiveness of collaborative care in treating anxiety among women remains limited (Grubbs *et al.*, 2015). Marcus *et al.* (2019) and Miller *et al.* (2020) described the unique features of collaborative care programs focused specifically on perinatal women and the efficacy of this model in addressing care gaps especially in areas with high health disparity; however, they did not evaluate the effectiveness of collaborative care in the treatment of anxiety in women. Curth *et al.* (2020) studied the effectiveness of the Collabri model (the Danish version of collaborative care) in the treatment of depression and anxiety in a sample with 64.8% women, of which 47.2% had a primary diagnosis of generalized anxiety disorder, but did not find differences in anxiety outcomes over time or between groups.

Collaborative care has been shown to be effective in a time-limited manner. The seminal study by Katon and others (1999) demonstrated the efficacy of collaborative care on depressive symptoms within the first 90 days of treatment. Since this study, other researchers have shown the time-limited effect of collaborative care treatment on reducing depressive symptoms. For example, Richards and colleagues (2008) studied the impact of collaborative care intervention on depressive symptoms in a three-month study. There was a significant reduction in depressive symptoms for the individuals receiving the intervention compared to usual care. Moreover, a meta-analysis showed that collaborative care was effective in improving depression rates in the short term, less than three months, compared to usual care (Sighinolfi *et al.*, 2014).

This study was designed to address the aforementioned gap in empirical knowledge by assessing the trajectory of anxiety symptoms among women within a Collaborative Care Model. Specifically, we sought to 1) assess if a collaborative care model instituted in women’s health settings is associated with reduced anxiety symptoms across the entire duration of treatment and within a short time period (ie, 90-days) and 2) evaluate what care factors (eg, primary diagnosis, duration of care, and use of psychotropic medications) may impact anxiety symptom outcomes among women in women’s healthcare settings.

## Methods

Secondary data analysis was used to test the stated questions using electronic records from Concert Health, a behavioral health medical group which partners with healthcare organizations to bring collaborative care to patients across sixteen states. Concert Health employs masters’ level clinicians and psychiatric nurse practitioners or physicians to provide care and consultation. Concert Health provides care and treatment virtually, offering the option to patients of video or telephonic care. The provision of collaborative care began with the original randomized controlled study as being predominantly telephonic follow-up to increase patient engagement and support follow-up on short-term goals established with patients and therefore was not as affected by COVID as other non-virtual models.

Data were obtained from the Concert Health electronic record of N = 617 women, across three women’s health centers served by Concert Health [1] during the period between January 2019 and December 2021. Data were de-identified using a unique id.

### Participants

This analytic cohort is composed of women served at one of three women’s healthcare sites (names not shared for privacy considerations) contracted with Concert Health in the following areas: (1) Avon Connecticut (N = 113), (2) Phoenix Arizona (N = 72), and (3) Saratoga New York (N = 34). Specific patient demographics are not available in the Concert Health patient registry and were therefore obtained from the most up-to-date US state and regional census data (United States Census Bureau, 2022). Overall, all three sites were predominantly Caucasian (NY: 91.5%, CT: 75.9%, and AZ: 72.9%) women. The sites varied by median income and percent of the population living below the poverty level (NY: $82 816, 6.0%; CT: $131 130, 3.4%; AZ: $57 459, 18%).

All patients in the analytic cohort were identified as needing behavioral health support through provider referrals and were enrolled in collaborative care services at Concert Health where they received an intake assessment with a behavioral care manager (BCM), reviewed by a psychiatric consultant, and follow-up evidence-based brief therapeutic interventions (eg, behavioral activation and motivational interviewing), care management with BCMs, and ongoing behavioral assessments (AIMS Center, 2022). Patients’ data were contained in a separate electronic record by Concert Health and were de-identified for analysis. Approval for this study was obtained from Adelphi University, Garden City, New York, IRB #081 021. Patients were given the option of telephonic or video care for each contact. Collaborative care, by design, is a predominantly telephonic or remote model, so this was consistent with the model despite COVID during a portion of the time of this study. The study utilized de-identified retrospective data, so individual consent from patients was not required or requested.

### Outcome measures

Data included dates of admission and discharge from Concert Health services. Patients are determined to be ready for discharge if they achieved symptom improvement and/or treatment goals. Anxiety symptoms were assessed using the Generalized Anxiety Disorder 7-item scale (GAD-7; Spitzer *et al.*, 2006). The GAD-7 is a validated scale that measures anxiety symptoms over the past two weeks. It is a 7-item Likert-type questionnaire that has a 4-point response scale (0 = Not at all, 1 = Several Days, 2 = Over half the days, 3 = Nearly every day). The range of possible scores is 0–21. Comparing the GAD-7 scores of women at the time of admission to collaborative care services with Concert Health and at the time of discharge, the study examined factors associated with a reduction in anxiety symptoms among women receiving collaborative care across clinic sites[LS5]. Responses were averaged to create a mean composite score at the time of admission. Additionally, scores were divided according to previously published clinical cutoffs (Plummer *et al.*, 2016) as minimal (0–4), mild (5–9), moderate (10–14), and severe (15–21) to assess changes in clinical severity from baseline scores to termination scores.

Primary DSM-V diagnosis by ICD-10 code was determined by an interview with the behavioral care manager and psychiatric consultant. The initial primary diagnoses were then further collapsed into three categories as follows: Anxiety Disorders (eg, Generalized Anxiety Disorder, Panic Disorder, Anxiety Disorder unspecified), Mood Disorders (eg, Major Depressive Disorder, Postpartum Depression, Bipolar Disorder, and Posttraumatic Stress Disorder), and Adjustment Disorders/Other. See Table 1.

**Table 1.** Descriptive statistics of GAD-7 scores by primary diagnosis

	*N*	Percentage (%)	Average baseline GAD by diagnosis	SD baseline GAD score	Average last GAD by diagnosis	SD last score GAD	Average between scores (days)	SD of time between scores (days)
Anxiety disorders (GAD, panic disorder, anxiety dx, unspecified, PTSD)	120	54.8	11.83	4.87	7.48	5.17	118.27	110.43
Mood disorders (MDD-single, MDD-recurrent, PPD, BPD, other depressive dx)	87	39.7	11.33	5.24	7.78	5.42	79.93	75.37
Adjustment disorder/Other	12	5.5	8.67	6.30	5.42	3.85	61	49.23
Total	219	100	11.47	5.14	7.57	5.35	100.03	96.72

Time between first admission GAD-7 score and last GAD-7 score (prior to discharge or last visit at Concert Health) was defined in days. Patients taking any psychotropic medications were coded in a binary manner with 1 representing the presence of medications. The specific drug names or dates of use were not available for analysis.

### Statistical methods

These data came from a larger cohort of individuals receiving collaborative care. There was no control group due to ethical considerations. Analysis of variance was conducted to determine if there were differences across the three collaborative care sites in the baseline anxiety scores. There was no significant difference in initial scores (F(218) = 0.67, *P* = 0.410); therefore, the collaborative care sites were collapsed into one group for final analyses and regression analyses. Prior to building correlation and regression models, data were checked for normality of distribution. The duration of time between scores was positively skewed (skewness = 1.5) and transformed using a square root transformation for analyses. A residual change score was calculated by regressing last scores on baseline GAD-7 scores. The predicted last scores were then subtracted from the observed last scores. The residual change score was used as the outcome variable in a linear regression model. Significance levels were set with an alpha of .05 and a 95% confidence interval. All analyses were run in the statistical software R, version 3.6.3 (R Core Team, 2021).

## Results

### Descriptive statistics

The sample included 617 individuals, 393 were excluded from the analyses due to missing data at the predictor level; this left a sample of 224 individuals in the analytic cohort. Upon initial analyses of the data, N = 5 outliers were removed from the final sample using the interquartile ranges resulting in a final sample of N = 219. Patients in the final analytic cohort (N = 219) carried primary ICD-10 diagnoses of Anxiety disorders (N = 120), Mood disorders (N = 87), and Adjustment Disorders/other disorders (N = 12). The average baseline GAD-7 score across all groups was 11.47 ± 5.14. Notably, patients with primary anxiety or mood disorders demonstrated moderate levels of anxiety symptoms at baseline – with those with anxiety disorders showing mean GAD-7 scores of 11.83 ± 4.87 compared to those with mood disorders having baseline mean GAD-7 scores of 11.33 ± 5.24 and those with adjustment disorders having baseline mean GAD-7 scores of 8.66 ± 6.30. Average time between baseline and final scores was 100 ± 96 days (Table 1).

Correlational analyses were run to assess the relationship between anxiety scores and predictors. There was a significant positive correlation between baseline and final anxiety scores (*r* = 0.57, *P* <0.001). There was a significant negative relationship between the duration of time between scores and anxiety scores at the time of discharge (*r* = −0.16, *P* = 0.016). Primary diagnosis was negatively associated with durations of time between scores (*r* = −0.19, *P* =0.005) (with anxiety disorders scored as 1), such that those with mood disorders or anxiety disorders were more likely to have a longer duration of time in the program compared to individuals with adjustment disorders/other. There was also a positive association between time between scores and patients on psychotropic medications (*r* = 0.23, *P* <0.001), such that the longer an individual was in the program, the more likely they were to be on psychotropic medications.

**Figure 1.**
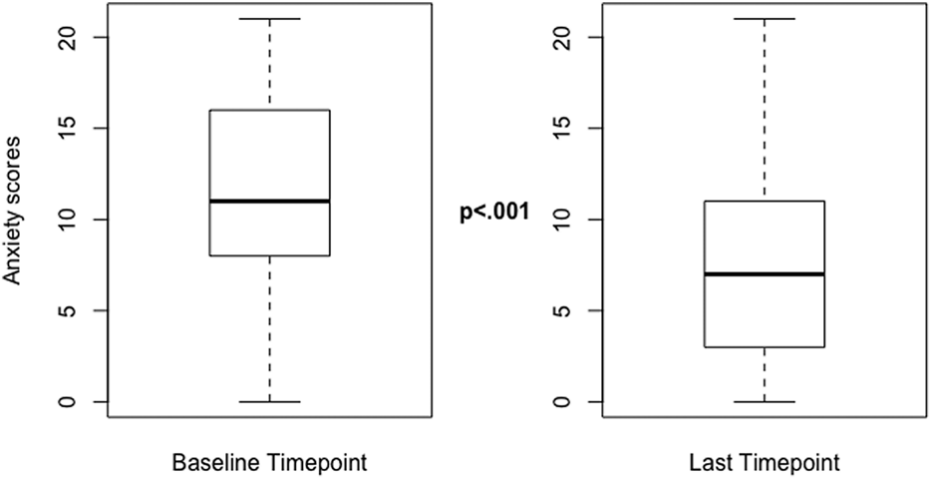
Median pre- and post-GAD-7 scores. Figure 1 demonstrates the median, interquartile ranges, and spread of these data. Paired Student’s *t*-test demonstrated a statistically significant difference between baseline GAD-7 scores and Last GAD-7 score (*P* < 0.001).

A paired Student’s *t*-test was done between anxiety baseline (M = 11.46, SD = 5.09) and last measures for collaborative care treatment (M = 7.48, SD = 5.18) and showed a statistically significant reduction in average anxiety (*t*(218) = 12.41, *P* < 0.001). Figure 1 depicts the mean, interquartile ranges, and significant difference between average anxiety symptoms at intake to the collaborative care program compared with average anxiety symptoms at discharge from it.

**Figure 2.**
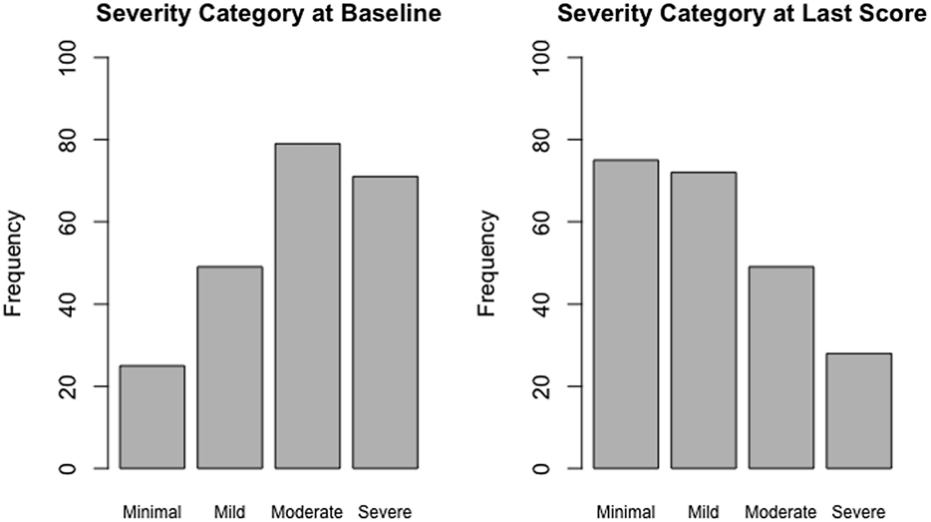
Baseline GAD-7 scores by clinical cutoffs in comparison to Last GAD-7 scores by clinical cutoffs. Figure 2 demonstrates the frequency of participants categorized using clinical cutoffs of GAD-7 scores at baseline and at last score.

At baseline, most patients scored in the moderate (N = 79) and severe (N = 71) range. This pattern was reversed at the termination of collaborative care services, with the majority of patients falling in the minimal (N = 75) and mild (N = 72) anxiety score ranges (Fig. 2).

### Regression model

**Figure 3.**
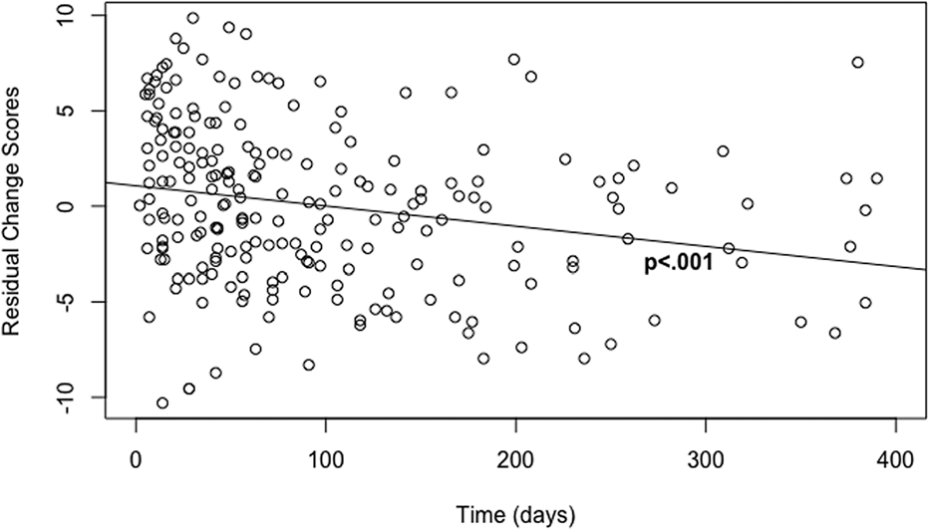
Reduction in GAD-7 scores from baseline to final score over time (days). Figure 3 demonstrates the regression model output of days enrolled in collaborative care and reduction in anxiety symptoms, using residual change scores (the last score accounting for baseline anxiety score).

Linear regressions, utilizing a residual change model and including only the significantly correlated variables (ie, duration of time, primary diagnosis, and psychotropic medications), were used to assess factors associated with the change in GAD-7 scores. There was a main effect for the duration of time between scores on the amount of change in anxiety symptoms among women within the collaborative care model (β = −0.28, SE = 0.06, *P* < 0.001), such that duration of treatment predicted a greater reduction of anxiety symptoms (Table 2 and Fig. 3). A paired Student’s *t*-test was then run within a subset of individuals using their baseline score and the last score prior to the 90-day mark to assess the impact of the 90-day benchmark on reduction in anxiety symptoms. There was a significant reduction in anxiety symptoms at the 90-day mark (*t*(218) = 10.58, *P* <0.001) between baseline scores (M = 11.46, SD = 5.09) and last score prior to the 90-day mark (M = 7.96, SD = 5.15). More research will have to be conducted to understand the relationship between anxiety symptom reduction and the 90-day benchmark. There was no statistically significant relationship between the presence of psychotropic medications or primary diagnosis on the change in anxiety symptoms.

## Discussion

This study was designed to help address the aforementioned gap in empirical knowledge by assessing anxiety level score changes in women’s health practices using a collaborative care model. The significant reduction in average anxiety scores from baseline to termination of collaborative care found in the current study expands on prior studies that have primarily focused on collaborative care in primary care settings and on depression outcomes (Archer *et al.*, 2012; Thota *et al.*, 2012). Our findings provide further evidence for the usefulness of collaborative care in treating not only depression but also anxiety (see Muntingh *et al.*, 2016 for a review), especially among women. Our study supports prior work by Curth *et al.* (2020) who assessed anxiety symptoms in an experimental trial with women receiving collaborative care or usual care. There were trends toward improvement in anxiety symptoms.

Additionally, because twenty percent of women use their obstetric/gynecological providers as their primary health care (especially during childbearing years) (Commonwealth Fund, 2020), assessing the efficacy of collaborative care in this care setting is critical in addressing care access (Miller *et al.*, 2012; Miller *et al.*, 2020). The effective treatment of anxiety in women has important healthcare implications as anxiety has been previously associated with increased obstetric complications (Kurki *et al.*, 2000; Uguz *et al.*, 2012; Fairbrother *et al.*, 2016; Mckee *et al.*, 2020), postpartum anxiety and depression, suicide, and multiple medical comorbidities (Sareen *et al.*, 2005; Kroenke *et al.*, 2007; Dennis *et al.*, 2017; Johnson, 2019; Spoorthy *et al.*, 2019). It is therefore of paramount importance that we are able to effectively address anxiety among women in both primary care and alternative care settings. Because this study was not an experimental trial, we cannot state that it ‘effectively’ reduced anxiety, just that it was associated with a reduction in anxiety. More studies will have to be done to evaluate this hypothesis.

Our study also demonstrated that the majority of anxiety scores reduced within 90 days of collaborative care treatment. This finding extends prior literature by studying how time-limited treatment of collaborative care can reduce anxiety symptoms. This is consistent with other studies showing the effectiveness of collaborative care in reducing depression symptoms within 90 days (Sighinolfia *et al.*, 2014; Richards *et al.*, 2008). The anxiety score reduction we observed within 90 days of enrollment in collaborative care is encouraging as it suggests that time-limited treatment may be associated with significant reductions in symptoms and supports the collaborative care model (AIMS Center, 2022). To our knowledge, no other studies have examined the value of time-limited treatment of collaborative care on anxiety symptoms nor among women’s health settings.

This study supports the benefits of collaborative care services and reduced anxiety scores in women’s healthcare settings and has important clinical and political implications. Clinical efforts to standardize screening for anxiety among women across care settings will help reduce undertreatment and facilitate access to care. Additional research on the implementation and effectiveness of collaborative care models across different care settings and populations is needed to inform care protocols and promote broader use of collaborative care models nationally and internationally. For example, does the collaborative care model approach effectively reduce anxiety symptoms among perinatal women? Can collaborative care improve suicide outcomes? Is traditional collaborative care effective in treating dual-diagnosis populations? Additional research demonstrating that collaborative care can be effectively used across women’s health sites will encourage more states to adopt collaborative care as part of their Medicaid fee schedules, promote the expansion of this care model, and potentially expand access to evidence-based behavioral health treatment for huge populations of women. With more research and adoption of this care model, insurance payers may be swayed to recognize women’s health sites as key collaborative care entities and reimburse them at rates equivalent to their primary care counterparts.

Although the findings of this study are significant, there are several limitations that warrant mention and need addressing in subsequent studies. The data set did not contain some important demographics (eg, age, race, ethnicity, education level, or socioeconomic status) which limit generalizability. Moreover, information was missing in the data set as to what psychotropic medications or concomitant dosage was prescribed, making it unclear as to the impact of psychotropic medications in reducing anxiety symptoms. The specific amount of care hours received by each patient could not be calculated and should be evaluated in future analyses; our findings, therefore, reflect the reduction of anxiety symptoms from enrollment to termination of collaborative care services. Only the primary diagnosis was assessed as a predictor for the reduction in anxiety symptoms. Some participants in our study did have comorbid diagnoses, which was not looked at in the scope of this study. Other studies have shown the effectiveness of collaborative care on comorbid diagnoses (Huffman *et al.*, 2014). Thus, future studies should look at the effectiveness of collaborative care on comorbid diagnoses in women’s health practices. There was also a substantial amount of data missing at the predictor level (ie, baseline or last GAD-7 score). Due to the limited nature of the dataset in terms of demographics, analyses were not done between those excluded and included. This study was only contained to women’s health practices. Since Grubb’s and others (2015) found a significant moderating effect of gender on the efficacy of collaborative care and anxiety symptoms, future studies should examine this. Another limitation was that pregnancy status was not recorded consistently, limiting assessment of whether pregnant and postpartum women with anxiety symptoms benefit from collaborative care or have different care needs. Another limitation that impacts the generalizability of the study is that the study design was retrospective and did not contain a control group (eg, that did not receive collaborative care). Therefore, any causal inference of collaborative care in the reduction of anxiety symptoms cannot be concluded. Because scores can naturally regress toward the mean, more studies will have to determine the efficacy of collaborative care by utilizing a control group. Lastly, shortly after the start of collaborative care treatment in this cohort, the COVID-19 pandemic began. Because of the proximity of the onset of the study to the COVID-19 pandemic, this could have impacted our results through spikes in anxiety symptoms due to the pandemic. This could limit the generalizability of the study for populations receiving treatment 3 years after the onset of the pandemic.

## Conclusion

Collaborative care is an effective care model that improves access to behavioral health care and mental health outcomes. While the vast majority of the literature has focused on the implementation of collaborative care services in primary care settings, additional research is needed to assess the care model’s relevance in alternative care settings, patient populations, and addressing different diagnoses. Our findings show that collaborative care is associated with a reduction in anxiety symptoms over a 90-day time period in women’s health practices. Future studies should evaluate the effectiveness of collaborative care more broadly to build a diverse research foundation to guide implementation and care protocols.

**Table 2.** Linear regression estimates for factors associated with changes in anxiety symptoms

Effect	Estimate	SE	95% CI		*P*-value
			LL	UL	
Intercept	2.60	0.68	1.25	3.95	<0.001
Primary diagnosis	−0.25	0.28	−0.79	0.29	0.366
Psychotropic medications	0.55	0.87	−1.17	2.27	0.530
Duration of time between scores	−0.28	0.06	−0.40	−0.15	<0.001

*Note.* Total *n* = 219. CI = confidence interval; LL = lower limit; UL = upper limit.
